# Lost to Follow-Up among Tuberculosis Patients during the Public-Private Mix Era in Rural Area of Indonesia

**DOI:** 10.4314/ejhs.v33i1.15

**Published:** 2023-01

**Authors:** Sri R Rahayu, Mustika S Susilastuti, Muhamad Z Saefurrohim, Mahalul Azam, Fitri Indrawati, Mamat Supriyono, Dani Miarso, Baiq D Safitri, Sabrina Daniswara, Aufiena NA Merzistya, Rizqi Amilia, Mustafa D Affandi, Nur Wahidah, Isbandi, Anggun D Wandastuti, Annisa K Laila, Zuyyinatun Muflikhah

**Affiliations:** 1 Public Health Department, Sport Science Faculty, Universitas Negeri Semarang, Indonesia; 2 Semarang City Health Office, Semarang, Indonesia; 3 Master of Public Health, Postgraduate, Universitas Negeri Semarang, Indonesia; 4 Gadjah Mada University Academic Hospital, Yogyakarta

**Keywords:** Health Facilities, Lost to Follow-Up, Tuberculosis, Indonesia

## Abstract

**Background:**

Indonesia's national Tuberculosis (TB) strategy is public-private mix (PPM). The PPM aims to treat patients who have lost sight during TB treatment as these patients are TB carriers and at risk of transmitting TB. The purpose of this study was to identify predictive factors for loss to follow-up (LFTU) among TB patients receiving treatment when the PPM was at place in Indonesia.

**Methods:**

The design of this study was a retrospective cohort study. The data used in this study was sourced from the Tuberculosis Information System (SITB) of Semarang which was recorded routinely during 2020–2021. Univariate analysis, crosstabulation, and logistic regression were performed on 3434 TB patients meeting the minimum variables.

**Results:**

The participation of health facilities in reporting TB during the PPM era in Semarang reached 97.6% consisting of 37 primary healthcare center (100%), 8 public hospitals (100%), 19 private hospitals (90.5%), and a community-based pulmonary health center (100%). The regression analysis reveal that the predictive factors of LTFU-TB during the PPM are the year of diagnosis (AOR=1.541; p-value=<0.001; 95% CI=1.228–1.934), referral status (AOR=1.562, p-value=0.007; 95% CI=1.130–2160), healthcare and social security insurance ownership (AOR=1.638; p-value=<0.001; 95% CI=1.263–2.124), drugs source (AOR=4.667; p-value=0.035; 95% CI=1.117–19.489).

**Conclusions:**

The PPM strategy in dealing with LTFU patients should focus on TB patients without Healthcare and Social Security Insurance and who receive TB treatment rather than program drugs.

## Introduction

Tuberculosis (TB) is an infectious disease that remains a major health problem worldwide. In 2019, the number of people diagnosed and confirmed TB cases reached 7.1 million globally (1). World Health Organization (WHO) estimates that there is a 2.9-million gap between the number of diagnosed TB cases and the number of notified cases due to the high unreported cases. One country that accounts for more than half of the global gap is Indonesia (10%) (2). The National TB strategy implemented in Indonesia and several other countries such as in Ethiopia is to increase case finding as the main focus of TB control (3–5).

WHO global policy for TB control is to involve all service providers through a Public-Private Mix (PPM) approach (1,6). The goal of PPM is to improve case detection and treatment success that contributes to missing cases (7,8). The target providers are not only private and corporate sector (hospitals or institutions, private practitioners), and voluntary sector (nongovernment organization or community-based organizations), but also public sector itself (many types of public providers such as general) and specialty hospitals, teaching hospitals, prisons, military-owned providers and others who have not joined the program (8,9). The case network is one of the PPM network's principles, namely the continuity of treatment of TB patients from referrals/transfers and the tracking of TB patients who are lost to follow-up (10).

This policy has also been adopted by the Government of Indonesia. Research evidence in India and Myanmar shows that PPM strengthens TB care and control (11). Research in Pakistan shows that Among the PPM approaches, general practitioners and non-governmental organization facilities achieve 94–95% treatment success; private hospitals achieved 82% success (12)

The PPM approach is to ensure equitable, quality, and sustainable access to TB services for those affected by TB (universal access) in ensuring TB patients' recovery. However, within the Indonesian context, private sector involvement remains low (9). According to Indonesia National Development Planning Agency, the private sector manages more than 50% of hospitals, provides 60% of outpatient care, and 43% of hospitals with inpatients (9,13). The Patient Pathway Analysis (PPA) study in 2017 revealed that 54% of the discovery and treatment of TB has been carried out by government health facilities,42% by the private sector, and another 4%. The proportion of TB cases from government hospitals and government clinics from the national target of 17% reached 16%, while from private hospitals the target of 23% was reached 22%, and from Clinics and independent practice doctor of the target of 1% achieved 2% (14). However, only 32% of cases are recorded, indicating that 68% of cases go unreported. Most of these missing cases are believed to be in the private sector and go unreported, even though some of them can receive both diagnosis and treatment at the same time.

The success of tuberculosis control in Indonesia can be described by three indicators. They are complete treatment rate (% complete rate), cure rate (% cure rate), and treatment success rate (% success rate). TB patients are therefore classified as cured, completed treatment, failed treatment, lost to follow-up, or died based on the outcome of their treatment. Lost to follow-up (LTFU) was defined as patients who received treatment for at least 4 weeks and the treatment was discontinued for more than eight consecutive weeks (15).

Previous studies reported that the factors for increasing LTFU in TB patients were negative attitudes towards treatment, limited social support, dissatisfaction with health services, and limited economic status (16). A study in Namibia reported that male gender, age group 15–24 years, treatment service providers, TB intensive phase patients, and living in border/transit areas were factors for LTFU in TB patients (17). A meta-analysis study reported that the high prevalence of LTFU in TB patients due to multidrug resistance, and the anatomic location of tuberculosis were significant factors (18). The significance of risk factors of LTFU (i.e. patient demographics, socioeconomic status, directly observed treatment, short-course (DOTS) programme, clinical covariates, TB treatment regimen and HIV co-infection) on LTFU has been contested across countries (17). One of the reasons for the development of acquired Drug Resistance Tuberculosis (DR-TB) is LTFU. Patients who are readmitted after LTFU are more likely to redevelop infectious active TB and are at higher risk of developing further drug resistant strains of tuberculosis (19,20). According to reports on the economic challenges of TB drug non-adherence, an estimated 52 MDR-TB patients lost to follow-up resulted in 5 patients developing XDR-TB, 3 newly infected MDR-TB and a new XDR-TB, and 3 deaths (18).

LTFU can increase the risk of clinical deterioration, treatment failure, and further complications in tuberculosis patients. Patients who discontinue treatment too early are one of the leading causes of treatment failure (18). The dropout rate is critical because low LTFU as a result of improved TB management will reduce re-treatment case by 10–20% in the coming years (10). This issue highlights the importance to study the predictors of LTFU during the PPM.

## Methods

**Study design and data source**: This study is an analysis of secondary data from the Tuberculosis Information System (SITB) through the TB03 form provided by the Ministry of Health. This system summarizes tuberculosis patient data and treatment monitoring. Research data was collected from 2020 to mid-2021. Respondents who were registered at SITB in 2020–2021 totaled 3400 respondents, 3434 respondents were examined for the final results of treatment, 384 were lost to follow-up, the rest were patients who died who were not included in the analysis.

**Dependent variable**: Patients whose tuberculosis treatment status was lost to follow-up were defined as discontinuing treatment for two or more consecutive months (8 weeks) for any reason without medical consent (18).

**Independent variables**: Independent variables that are fully recorded in SITB and can be included in the final analysis include the variables of Year Diagnosed, Insurance Ownership, Standard of Treatment, Gender, Occupational Status, Place of Residence, Close Contact Examination, Referral Status, Type of Diagnosis Enforcement, Type of TB, Patient Referral Status, Diabetes Mellitus Status, HIV Status, Drug Source.

The year diagnosed is the year when the respondent first received a TB diagnosis through molecular rapid tests, X-rays, and Mantoux which were classified into 2020 and 2021 (January – July); Insurance ownership is classified into having insurance and not having insurance; Standards of Treatment are respondents who are given treatment in accordance with the National Guidelines for Medical Services for Tuberculosis Management; Gender is classified into male and female; Employment status is classified into working and not working; Place of residence is categorized into Semarang City and Outside Semarang City; Close contact examination is classified into close contact examination or no close contact examination; Referral status is categorized into referral patients or patients who come alone to health services for TB tests; Methods of diagnosis are categorized into diagnosis of TB clinically or bacteriologically; The type of TB is classified into pulmonary TB or extrapulmonary tuberculosis; Patient status is categorized into whether the patient failed treatment or relapsed or new patient; Diabetes Mellitus status was grouped into positive TB patient Diabetes mellitus or negative TB patient diabetes mellitus; HIV status was categorized into TB patients who were HIV positive or TB patients who were HIV negative; and Sources of Drugs that are categorized into program drugs (free) or outside the program (own costs).

**Statistical analysis**: The data is presented in frequency and percentage based on a LTFU status. Chi-square analysis was performed to determine the relationship between the independent and dependent variables. P-value <0.05 was considered statistically significant. The independent variable, which has a p-value lower than 0.25, is included in the multivariable analysis. We analyzed the final model using Binary Regression Logistics Backward LR. All analyzes were performed by SPSS 22.0 (IBM Corporation, NY, USA).

**Ethical approval**: The Health Research Ethics Committee of Universitas Negeri Semarang has reviewed and approved the protocol by issuing a letter numbered 095/KEPK/EC/2021.

## Results

**Figure 1:**
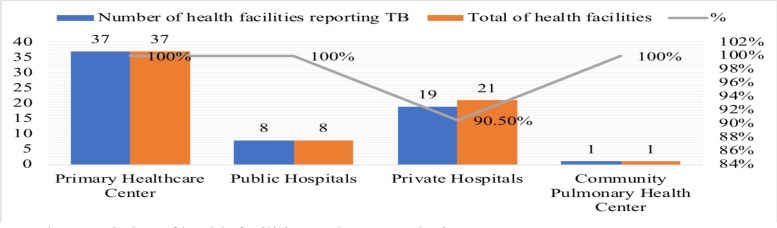
Characteristics of health facilities at the research site

Health facilities in Semarang City consist of primary healthcare center, public and private hospitals, community pulmonary health center, as well as independent practice doctors and clinics which are reported through primary healthcare center and hospitals as referral health facilities. All 37 primary healthcare center in Semarang (100%) participated in reporting and recording TB cases. Likewise, 8 public hospitals and a primary healthcare center, all of them (100%) participated in the reporting and recording TB cases. However, out of 21 private hospitals in Semarang, only 90.5% private hospitals participated in reporting and recording TB cases (Fig. 1).

Characteristics of respondents based on Tuberculosis Information System (SITB) data are presented in Table 1. The average age of TB patients is 34.2 years old (SD: 28.9), with male patients (53.9%) outnumbers female patients. Most of them live in Semarang (77.4%), while the rest live in other neighboring cities. As many as 81.8% of pulmonary TB patients and their sis were extra pulmonary TB patients. From the data analyzed, many TB patients have comorbid diseases, 8.8% of TB patients had positive Diabetes Mellitus status while 1.5% of TB patients had HIV positive.

**Table 1:** Characteristics of Tuberculosis patients in Semarang City based on data from Tuberculosis Information System (SITB) (N=3434)

Characteristic	Frequency (n)	Percent
**Age (mean; SD)**	34.2;	28.9
**Year of Diagnosis**		
2020	2282	66.5
2021	1152	33.5
**Sex**		
Male	1851	53.9
Female	1583	46.1
**Employment Status**		
Employed	2348	68.4
Unemployed	1086	31.6
**Residence Status**		
Out of town	775	22.6
In the town	2659	77.4
**TB Types**		
Pulmonary TB	2810	81.8
Pulmonary Extract TB	624	18.2
**Diabetes Mellitus Status**		
Positive	301	8.8
Negative	3133	91.2
**HIV Status**		
Positive	51	1.5
Negative	3383	98.5
**Patient Status**		
Treatment failure	120	3.5
Relapse	49	1.4
New Patients	3265	95.1

Patient characteristics, including age (p-value=<0.001), year of diagnosis (p-value =0.001; RR=0.730; 95% CI: 0.603- 0.883), gender (p-value=0.007; RR=1.305; 95% CI=1.076–1.584), employment (p-value=0.013; RR=1.314; 95% CI=1.058–1.631), residence (p-value=<0.001; RR =1.503, 95% CI=1.228–1.840), type of TB (p-value=0.013; RR=1.418; 95% CI=1.072–1.874), Diabetes Mellitus (p-value=<0.001; RR = 1.852; 95% CI = 1.438–2.385), and patient status (p-value=0.016; RR = 1.537; 95% CI = 1.018–2.321). These characteristics were statistically associated with the incidence of LFTU during TB treatment. In addition, healthcare and social security insurance ownership (p-value=<0.001; RR 0.621; 95% CI = 0.495–0.779), standard treatment (p-value=<0.001; RR=1.774; 95% CI=1.389–2.265), close contact examination (p-= value=0.018; RR=1.859; 95% CI=1.089–3.174), referral status (p-value=<0.001; RR=0.574; 95% CI =0.432–0.764), diagnosis method (p-value=0.004; RR=1.344; 95% CI= 1.100–1.643), drugs sources (p-value=0.045; RR:0.285; 95% CI=0.073–1.117) and had a positive significant relationship with the LFTU during TB treatment during the PPM (Table 2).

**Table 2:** Cross-tabulation predictive factors of lost to follow-up status during tuberculosis treatment in public-private mix era

	Final Result of Treatment						
				
	LTFU		Recovery		RR	95% CI	*p*-Value
				
Variable	n	%	n	%			
**Age (mean; SD)**	384	11.2	3050	88.8	-	6.405–10.825	<0.001
**Year of Diagnosis**							
2020	227	9.9	2055	90.1	0.730	0.603–0.883	0.001
2021	157	13.6	995	86.4			
**Healthcare and Social Security** **Agency ownership**							
Do not have	88	7.9	1024	92.1	0.621	0.495–0.779	<0.001
Have	296	12.7	2026	87.3			
**Treatment Standard**							
Non-standard	64	18.4	284	81.6	1.774	1.389–2.265	<0.001
Standard	320	10.4	2766	89.6			
**Sex**							
Male	232	12.5	1619	87.5	1.305	1.076–1.584	0.007
Female	152	9.6	1431	90.4			
**Employment Status**							
Employed	284	12.1	2064	87.9	1.314	1.058–1.631	0.013
Unemployed	100	9.2	986	90.8			
**Residence**							
Out of town	117	15.1	658	84.9	1.503	1.228–1.840	<0.001
In the town	267	10.0	2392	90.0			
**Close Contact Examination**							
No	371	11.5	2853	88.5	1.859	1.089–3.174	0.018
Yes	13	6.2	197	93.8			
**Referral Status**							
Referral	50	7.0	660	93.0	0.574	0.432–0.764	<0.001
Non-referral	334	12.3	2390	87.7			
**Diagnose Method**							
Clinical	255	12.5	1789	87.5	1.344	1.100–1.643	0.004
Bacteriological	129	9.3	1261	90.7			
**TB Types**							
Pulmonary TB	332	11.8	2478	88.2	1.418	1.072–1.874	0.013
Pulmonary Extract TB	52	8.3	572	91.7			
**Patient Status**							
Treatment failure	20	16.7	100	83.3	ref.	ref.	0.016
Relapse	10	20.4	39	79.6	0.817	0.413–1.616	
New Patients	354	10.8	2911	89.2	1.537	1.018–2.321	
**Diabetes Mellitus Status**							
Positive	58	19.3	243	80.7	1.852	1.438–2.385	<0.001
Negative	326	10.4	2807	89.6			
**HIV Status**							
Positive	9	17.6	42	82.4	1.592	0.873–2.902	0.14
Negative	375	11.1	3008	88.9			
**Drug Source**							
Apart from program	2	3.2	60	96.8	0.285	0.073–1.117	0.045
From program	382	11.3	2990	88.7			

*P-value<0.05 = significant

The regression analysis was carried out by multivariate analysis (see Table 3). It revealed 4 key variables which became the predictive factors of lost to follow-up status during tuberculosis treatment during the PPM. The variables are year of diagnose (AOR=1.541; p-value=<0.001; 95% CI=1.228–1.934), referral status (AOR=1.562, p-value=0.007; 95% CI=1.130–2.160), healthcare and social security insurance ownership (AOR=1.638; p-value=<0.001; 95% CI=1.263–2.124, and drugs sources (AOR=4.667; p-value=0.035; 95% CI=1.117–19489).

**Table 3:** Logistic Regression predictive factors of lost to follow-up status during tuberculosis treatment in public-private mix era

Variable	*P*-Value	Adjusted OR	95%CI
Year of Diagnosis	<0.001	1.541	1.228–1.934
Referral Status	0.007	1.562	1.130–2.160
Healthcare and Social Security Agency Ownership	<0.001	1.638	1.263–2.124
Drug Source	0.035	4.667	1.117–19.489

*P-value<0.05 = significant

## Discussion

The national strategy of TB Control Program aims at providing universal access to quality TB services through a systematic Find Cure Until Heal (TOSS) activity for all TB patients supported by active participation of health care providers both in public and private sectors (6,10,21). The PPM involves all health care facilities to expand TB patient services and the continuity of a comprehensive TB control program. One of the objectives is to prevent LTFU patients during TB treatment (10,21). Most health facilities in Semarang, both private and public, have contributed to TB reporting. This contribution was supported by the fact that TB patients have good knowledge on signs and symptoms of TB, transmission of TB and healthcare seeking behavior of TB (22). In practice, government health facilities (hospitals and primary healthcare center) have reported more cases than private hospitals.

The logistic regression analysis revealed three key factors that influenced the work of LTFU in tuberculosis patients during the PPM period: the patient was referral, the patient did not have any healthcare and social security insurance, and the medication received was not a program drug. According to this study, the most significant factor influencing the incidence of LTFU during the PPM period is patients who receive drug sources other than the program, with 4.6 times probability. These findings suggest that patients tend to use complementary medicine. Previous research has shown that in general, Asians use complementary medicine in addition to conventional medicine (23). In addition, community's influence plays a strong role in TB medication in Asia. Accessibility, tradition or belief, and feelings of dissatisfaction with conventional drugs are all factors that encourage the use of alternative drugs. Another factor that affects LTFU with 1.5 times probability is referral patients (24). Research in Pakistan shows that patients who undergo treatment at referral health facilities and become referral patients are more likely to experience LTFU before starting TB treatment (25). This is due to the distance between the patient's residence and the facility. The greater the distance, the higher the LTFU rate, particularly for patients living outside the city (26).

Because treatment is not cheap and takes a long time, health insurance is essential for TB patients in Indonesia, who are mostly from lower-middle-class families. TB patients' treatment costs more because they must pay for co-morbidity medication, transportation, and accommodation (27). Furthermore, the indirect costs of TB treatment the patients have to endure are reduced income or a lower proportion of household income, which can lead to deeper poverty. According to previous research, the costs incurred when a person does not work while on treatment account for 67% of the total costs incurred by TB patients (28).

The results of the study, which found that patients without health insurance had a 1.6 times greater likelihood of missing treatment, were relevant to previous studies because having health insurance ensures that TB patients do not incur personal costs for care and treatment until they are declared cured. The availability of health insurance is critical, particularly in cases of TB with complications or additional conditions such as diabetes, hypertension, impaired kidney function, pregnancy and lactation, or other diseases that necessitate additional examination and treatment. Patients without health insurance must still pay for additional examinations, hospitalization, or other drugs not covered by the government's TB program. Due to the high costs, TB patients, particularly those without health insurance, are more likely to be absent or to discontinue treatment (29).

LTFU factors in TB patients should be better understood for a better understanding of treatment adherence challenges, especially during the PPM initiative. As a result, we recommend a qualitative study to assess other factors that increase the risk of LTFU that are reviewed in cross-sectoral services and support, particularly private health facilities in the PPM period, and how health workers treat LTFU patients. One limitation of this study is that we assessed LTFU using electronic records at the SITB rather than actively tracking patients. Furthermore, there is no access to a list of LTFU patients at facilities located outside of Semarang City, so it is possible that some TB patients have transferred treatment to locations outside of the city but are still classified as LTFU. However, as these patients represented only 11.2% of all patients in this study, this factor is unlikely to have had much effect on the overall outcome. Another limitation is that because the data is secondary, we were unable to determine the exact factors for LTFU from the patient's perspective. Despite these limitations, this study extends our understanding of the factors that contribute to LTFU during TB treatment during the PPM initiative. The TB control program manager can use this information as key reference to optimize the implementation of PPM in the context of TB control.
